# Cancer resource center of the desert patient navigator program: removing financial barriers to access to cancer care for rural Latinos

**DOI:** 10.1186/2193-1801-2-15

**Published:** 2013-01-18

**Authors:** Diana Peacher, Helen Palomino, Eunjeong Ko, Susan I Woodruff

**Affiliations:** 1 Cancer Resource Center of the Desert, 444 So.8th Street Ste. B-3, 92243 El Centro, CA USA; 2 Cancer Resource Center of the Desert, 444 So.8th Street Ste. B-3, 92243 El Centro, CA USA; 3 San Diego State University, School of Social Work, 5500 Campanile Drive, 92182-4119 San Diego, CA USA; 4 Center for Alcohol and Drug Studies, San Diego State University, School of Social Work, 6386 Alvarado Court Ste 224, 92120 San Diego, CA USA

## Abstract

**Background:**

Health disparities in cancer mortality for racial/ethnic minorities is a public health concern. Financial barriers are the major factors preventing cancer patients from accessing treatment in a timely manner. This article describes the characteristics of the Cancer Resource Center of the Desert (CRCD) Patient Navigator Program (PNP) in the rural underserved US-Mexico border region of the Imperial Valley. Financial navigation services and the insurance conversion process for cancer treatment are described.

**Findings:**

CRCD data from 2010 to 2011 were analyzed to report the characteristics of cancer patients, focusing on insurance status changes. Eighty-one to 87% of the patients served were Latino/Hispanic. A case scenario is presented to depict the financial navigation process in converting the patients’ insurance status. Among the total samples, about 7% (n=32) in 2010 and 16% (n=68) in 2011 were in need of health insurance assistance upon their intake. Financial navigators successfully converted virtually all non- or inadequately-insured rural cancer patients to better insurance status.

**Conclusion:**

Financial concerns are a significant thread that runs throughout the diagnostic, treatment, and post treatment journey of cancer patients. The complicated nature of patients’ circumstances and medical systems often hinders the patients going through the insurance conversion process. PNP plays a critical role in bridging the gap between patients and medical systems thus promoting cancer treatment access for this vulnerable population.

## Introduction

Health disparities in cancer mortality for racial/ethnic minorities is a public health concern (American Cancer Society 2009). Further, minority individuals living in rural areas face additional unique challenges related to health care access, including limited health care facilities, physical distance, low socio-economic status, and rural or cultural practices associated with health behaviors and beliefs (Beyer et al. 2011; Eberhardt and Pamuk 2004). The stage of cancer at diagnosis also differs by urban/rural status, with individuals who live in rural areas more likely to be diagnosed with cancer at a later stage (Schootman et al. 2003) and less likely to utilize cancer screening (Coughlin et al. 2002) than people residing in urban regions. Latinos, in particular, may face multiple barriers to timely cancer care due to financial, structural, personal, and cultural factors (American Cancer Society 2009). Latinos are more likely to work in low wage jobs (e.g., agricultural and domestic work) and are less likely to have health insurance than any other ethnic group (American Cancer Society 2009). Among subgroups of Hispanic/Latinos, Mexican Americans have a higher percentage of poverty, less education, and a greater likelihood of no regular source of medical care (American Cancer Society 2009). Inequities and disparities in access to health care may increase the cancer burden for this at-risk population, and heighten the need for public health efforts to reduce these barriers (American Cancer Society 2009).

### Cancer navigation program

The concept of the Patient Navigation Program (PNP) was first introduced in the 1990s by Dr. Harold Freeman as an intervention to reduce barriers among African American and Latino breast cancer patients in Harlem, NY (Freeman 2006). The program aims to reduce the barriers that impede cancer patients’ access and utilization of health care prevention, diagnostic and treatment services, and aims to ensure that these are received in a timely manner (Robie et al. 2011), thus filling the gap between “discovery and delivery” of health care services.

Freeman (2004) indicated that the major determinants that contribute to this gap include culture, poverty, and social injustice. Barriers associated with these factors contribute to the persistent nature of health disparities. Cultural barriers found in the scholarly literature include health literacy, language issues, and cultural beliefs about cancer and cancer care (Freeman and Chu 2005). Poverty-related barriers include financial deficits, poor communication between health care providers and patients, transportation concerns, and fragmented medical care (Freeman and Chu 2005). In particular, financial barriers are the major factors preventing cancer patients from accessing treatment in a timely manner (Lin et al. 2008). Social injustice refers to structural barriers including racial discrimination and bias that disproportionately affects access to health services for certain populations (Freeman and Chu 2005). These factors are interrelated and taken as a whole, can significantly affect a range of health care services and outcomes, including prevention, post treatment management, and even mortality (Freeman and Chu 2005; Freeman and Vydelingum 2006). Racial/ethnic minority status, combined with these barriers, places these individuals at a greater risk for experiencing health disparities.

A number of previous studies found positive outcomes associated with the Patient Navigation Program. It has been shown to be beneficial in increasing patients’ cancer knowledge (Schlueter et al. 2010), decreasing emotional distress (Davis et al. 2009; Ferrante et al. 2008; Schlueter et al. 2010), and increasing emotional support (Carroll et al. 2010; Schlueter et al. 2010). The program also is noted to have increased patients’ completion of cancer screenings (Chen et al. 2008; Jandorf et al. 2005), timeliness to diagnosis after abnormal cancer screenings (Ferrante et al. 2008), and increased adherence to follow-up treatments (Ell et al. 2007) by providing transportation services, education, assistance with appointments, and coordination of services. The effects of PNP are greater when navigators are community members (Steinberg et al. 2006). Community member navigators are resourceful in recognizing and addressing culture related barriers (e.g., health beliefs, language barrier) and play an important role in connecting communities and facilitating dialogues between patients and members of the health care system. Hence, there has been a growing recognition that the development of a PNP should address cultural diversity and competence (Han et al. 2009; Petereit et al. 2008; Steinberg et al. 2006).

### Cancer resource center of the desert (CRCD) patient navigator program (PNP)

The Cancer Resource Center of the Desert (CRCD) was established in Imperial County in September, 2006, to educate, guide, and empower the cancer patient and family through the cancer journey in a non-hospital based setting. Imperial County, California, is a desert region that is heavily based on agriculture. It is located in the southeast corner of California on the US/Mexico border. Over 75% of the population in Imperial County is Hispanic/Latino, many of whom are first generation immigrants. It is the poorest county in the State and has the highest unemployment rate at 28% (Imperial County Public Health Department 2008). Not surprisingly, statistics indicate that in 2005, 34% of Imperial County adult residents were uninsured all or part of the year as compared to 25% statewide (Imperial County Public Health Department 2008). Compared to the overall state, about twice as many Imperial County adult residents 19–64 years of age are Medicaid beneficiaries (Imperial County Public Health Department 2008). The region is vastly medically underserved (Office of Statewide Health Planning and Development OSHPD 2010). For example, although the leading cause of death in Imperial County is cancer (American Cancer Society 2009), cancer resources are extremely limited in that there is only one cancer center in the 4,500 square mile county.

Due to the unique characteristics of the area and population, the CRCD established a PNP as part of its services. The CRCD PNP is unique in that it has been designed to address specific needs and cultural barriers in the region. Some of the specific aspects of cancer care that the PNP must address include: (a) bi-national healthcare, insofar as many of those in Imperial County are initially diagnosed and receive some treatment in Mexico, (b) lack or inadequate insurance to pay deductibles, co-pays, and insurance premiums, (c) lack of transportation to and from diagnostic and treatment visits both within the county and out-of-county, (d) lack of culturally appropriate care (e.g., bilingual health care workers), and (e) geographical and climatic extremes including long distances from care and temperatures that can well exceed 100°F much of the year. For any or all of the foregoing reasons, patients often skip, delay, or completely forego effective cancer treatment.

Although CRCD PNP provides patient education, counseling, case management, and community outreach and education, it has found that that one of the most critical needs is “financial navigation.” While financial navigation can include assistance and advice about personal budgets, taxes, financial strategies, and credit options, improved insurance coverage has been the most successful financial approach for this population to relieve the monetary stress or hardship associated with cancer treatment. Because no insurance or inadequate insurance profoundly affects patients’ timely treatment, the CRCD PNP focuses resources on mitigating or eliminating potential barriers to treatment caused by lack of adequate insurance coverage. This manuscript describes the CRCD PNP clients and the results of its financial navigation efforts.

### CRCD PNP program description

The CRCD PNP is focused on intervention for the individual across the cancer continuum, supporting clients who are newly diagnosed as well as those already in treatment. Although it is hard to estimate the number of patients in the Imperial County catchment area who would need CRCD services, the American Cancer Society 2009 estimates about 500 new cancer cases each year in Imperial County. CRCD PNP has served over 1,300 unduplicated patients since September 2006.

Funding for CRCD is provided primarily through fundraising activities, donations through the Combined Federal Campaign (Federal United Way), and local foundation grants all totaling $186,634 in 2011. Office space and utilities are provided in-kind by the local Pioneers Memorial Hospital. Cancer patients and their families are referred by word of mouth from other cancer patients in treatment, as well as referrals from the local radiation clinic, the two local hospitals, local home health and community agencies, and the County Department of Social Services.

The only eligibility requirement for CRCD services is an active cancer diagnosis.

Patients who report experiencing barriers to cancer care are all eligible for enrollment in PNP. Five types of barriers to cancer care are assessed: (1) Financial/insurance barriers which refer to financial need or no/inadequate health insurance benefits to cover treatment costs; (2) Transportation barriers, which refer to a need for public and/or private transportation assistance; (3) Communication/cultural barriers, which include language barriers, health literacy, and communication issues with health care providers related to diagnosis and treatment; (4) Physical needs which include, assistance with housing, food, prosthesis, and extended care; and (5) Other needs such as referral for mental health counseling.

CRCD’s PNP is staffed by two full time navigators. Both Patient Navigators are bilingual speakers in English and Spanish; one is certified in the Patient Navigator program by the Harold P. Freeman Institute, New York. Both hold degrees in the field of Human Services or Social Work, and each have six years of experience in cancer navigation.

Upon arriving at the CRCD office, patients are greeted and assisted by staff. The first visit typically involves using an Intake Form to assess the client’s needs, including their health insurance status.

If financial navigation is needed, steps to convert non-insured or inadequately insured to adequate insurance status begins immediately. Examples of issues that navigators consider in assisting patients for insurance conversion include:

1. Is the patient insured? If not, do they qualify for any MediCal (California’s Medicaid program) category?
2. If insured, what is the ‘cap’ for the patient’s coverage? Is the coverage employer- provided? Will the patient be able to keep the coverage should he/she become disabled for longer than what the employer allows?
3. Is the patient covered under the Imperial County Medical Services Program (CMSP)? This is a potential barrier to any treatment outside of Imperial County, as there are no neighboring counties that accept this insurance. This coverage is also very limited with many diagnostic tests and medications not covered.
4. If the patient has CMSP or state MediCal, what is his/her share of cost? This monthly share of cost can range from a few dollars to thousands of dollars per month. Navigators assist with the application and mitigation of the share of cost.
5. Is the patient eligible for MediCal for the Aged, Blind, and Disabled, special programs that would fully cover diagnostic tests and procedures?
6. Does the patient have state MediCal under a “family” code which is contingent upon the family income and minor children? Navigators explore whether it would be advantageous for patients to apply for MediCal under a different code. By doing this, any change to the family does not change their MediCal status.
7. Does the patient qualify for California’s BCCTP (Breast and Cervical Cancer Treatment Program) or IMPACT (Improving Access, Counseling, and Treatment for Californians with Prostate Cancer)? These are special state MediCal programs that serve as safeguards for cancer patients who are uninsured or underinsured.

As an example of the types and breadth of financial navigation provided by the CRCD PNP, we provide a description of an actual patient. This patient had employer-provided health insurance but its coverage would cap out during her hospital stay when she was referred to CRCD. Mrs. P., a married non-English speaking, immigrant farm worker of Mexican descent was admitted to the emergency room of the local Imperial Valley hospital with severe abdominal pain. While hospitalized, it was found that she had stage III colon cancer. The social worker referred the patient to CRCD for psychological counseling, cancer education, and financial navigation services. CRCD followed up by contacting the patient while she was in the hospital, and family members came to the CRCD office to initiate enrollment in CRCD PNP navigation. Upon discharge from the hospital, Mrs. P. met with CRCD staff for further needs assessment. The navigator identified multiple issues that could impact the patient’s course of treatment: (a) both the patient and her husband had limited knowledge about her cancer diagnosis, the course of treatment, and her prognosis; (b) her insurance coverage was limited and had capped while she was in the hospital; and (c) she would no longer be able to work, thus changing the family income and ability to pay out of pocket expenses. With the given circumstances, the imminent issues Mrs. P. faced were: the continuation of her medical follow-up and treatments including seeking consultations from specialists in another county; completion of diagnostic tests; adherence to cancer treatment; and medication management.

First Step: Based on the CRCD financial navigator’s assessment of the patient’s income, assets, and property holdings, the financial navigator facilitated an application for MediCal. By applying before the end of the month, MediCal coverage extended retroactively to the first day of the same month, thereby acting as secondary insurance until the primary’s cap was reached. Within 48 hours of the submission of the application, Mrs. P. was found to qualify for County MediCal (CMSP). Regardless, insurance barriers remained. CMSP is limited to in-county coverage only and, while Mrs. P. was deemed to be medically needy, she had a $700 monthly share of cost or co-pay because the family income exceeded the program limit by $700. This monthly out of pocket expense must be paid first, before CMSP pays the provider.

Second Step. Given the severity of Mrs. P’s cancer diagnosis and inability to resume her work as a farm worker, CRCD financial navigators concurrently facilitated an application for the special MediCal category for the Aged, Blind, and Disabled, and also for Social Security Disability Insurance (SSDI).

Third Step. During the process of the MediCal application, Mrs. P’s cancer stage changed to Stage IV. Financial navigators immediately forwarded the new medical information to a local MediCal eligibility analyst for the purpose of establishing presumptive State MediCal Status (if approved, the patient would now be able to attend medical appointments to out-of-county medical providers as referred by the local oncologist). Because the patient was considered terminal, the patient was given *presumptive* State MediCal by the County. Since it is *Presumptive only,* the share of cost continued to be charged to the patient until a final determination was made by the state. Once the state made the determination, the budget was re-worked to accommodate a higher need threshold. The end result was a zero share of cost for the patient. Another outcome was that the Mrs. P. was eligible to receive reimbursement for the previous share of cost payments. With this changed insurance status, the patient (and CRCD financial navigators) could now contact medical billers to mitigate the over $80,000 in medical bills incurred since Mrs. P.’s hospitalization. The entire insurance conversion process took about 6 weeks from the first date of financial navigation at CRCD PNP until MediCal was approved.

## Methodology

### Patient navigation data

To describe the characteristics of patients and the outcome of their financial navigation, data were obtained from the Patient Navigation Vista Share Outcome Tracker database. This database contains patients’ sociodemographic characteristics, cancer related information, and barriers and needs to cancer treatment. Intake was conducted via face to face interview by staff members using a structured intake form. Once the intake was completed, all patients’ information was entered into the Vista Share database. Data from 2010 and 2011 were extracted, converted to an Excel file by a staff member at CRCD, and patient identifying information (e.g., name, address, contact number) was removed. The data was then converted into the SPSS program. This study was approved by the Institutional Review Board (IRB# 691078).

## Results

### Characteristics of patients referred to the CRCD

To describe the type of patients referred to the CRCD, Table 1 shows the distribution of several background variables collected on the Intake Form for 457 patients in 2010 and 428 patients in 2011. Socio-demographic information of the patients was similar in both years. About 70% of the patients were 50 years old and about two-thirds were female. The great majority of patients (81% in 2010; 86.7% in 2011) were Latino, with 10% in 2011 and 15% in 2011 reporting being White non-Latino. About two thirds (61% in 2010; 63% in 2011) identified Spanish as their primary language. With regard to the types of cancer diagnoses, about one third of the patients were diagnosed with breast cancer, followed by blood-related cancers and prostate cancer in both years.

**Table 1:** **Characteristics of cancer patients receiving services at the CRCD PNP in 2010 and 2011**

Participant characteristics	n (%)	
	2010 (n=457)	2011 (n =428)
**Age**		
0–19	11 (2)	11 (2.6)
20–39	23 (5)	25 (5.8)
40–49	45 (10)	64 (15.0)
50–59	95 (21)	105 (24.5)
60–69	104 (23)	94 (22.0)
70 and older	136 (30)	102 (23.8)
Unknown age	43 (9)	27 (6.3)
**Gender**		
Female	276 (60)	266 (62.2)
Male	181 (40)	162 (37.8)
**Race/Ethnicity**		
Latino	370 (81)	371 (86.7)
White non-Latino	69 (15)	42 (9.8)
African American	10 (2)	10 (2.3)
Asian/Pacific Islander	7 (2)	2 (0.5)
Other	1 (0.1)	3 (0.7)
**Major Types of Cancer**		
Breast	142 (31)	141 (32.9)
Blood^a^	64 (14)	56 (13.2)
Prostate	49 (11)	45 (10.5)
Colorectal	44 (10)	38 (8.9)
Lung	30 (7)	21 (4.9)
Pancreatic	12 (3)	15 (3.5)
Cervical	11 (2)	12 (2.8)
Other/unknown	105 (23)	100 (23.4)
**Primary Language**		
English	178 (39)	157 (37)
Spanish	279 (61)	271 (63)
**Needed financial/insurance navigation**	32 (7.0)	68 (15.9)
**Insurance Status prior to PNP (those who were in need of financial navigation)**		
Uninsured	15 (3.3)	32 (7.5)
Partially Insured	11 (2.4)	25 (5.8)
Fully Insured, but had limited coverage	6 (1.3)	11 (2.6)

^a^ Includes Leukemia, Hodgkins Lymphoma, Non-Hodgkins Lymphoma, and Multiple Myeloma.

### Insurance conversion

In 2010, the CRCD served 457 patients, about half (n=241) of which were newly referred patients. Among those, records indicate that 32 patients (7%) reported a financial barrier and were in need of financial navigation (see Table 1). There were 37 insurance changes made for these patients. However, this is an underestimated number of patients receiving financial navigation in 2010, because electronic tracking of this information was not implemented until the end of 2010. Fifteen of the 32 were initially uninsured. Among them, 12 were converted to full insurance coverage (e.g., the BCCTP program), and three were converted to partial coverage such as through the County Medical Services Program. For the 11 who had partial insurance, all were converted to full insurance. Six patients with full insurance coverage were converted to full insurance with better benefits which typically meant more treatment options and no premium or co-payment.

**Figure 1:**
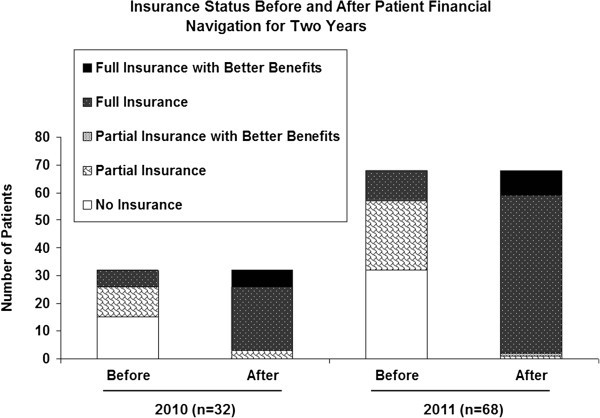
**Insurance Status Before and After Patient Financial Navigation for Two Years.**

In 2011, the CRCD served 428 patients, 221 of which were newly referred. Among the total patients in 2011, 68 patients (15.9%) reported facing financial barriers and were in need of financial navigation (see Table 1). There were 83 insurance changes made for these patients. Thirty-two patients were initially uninsured; 31 of them became fully insured as a result of the CRCD PNP, and one gained partial coverage. Among 25 patients initially partially insured, 24 were converted to full insurance, and one was converted to partial insurance with better benefits. Among those 11 patients initially with full insurance, nine were converted to full insurance with better benefits. Figure 1 graphically shows the change in groups’ needing financial navigation in 2010 and 2011. Relatively darker shading indicates a more positive status in insurance coverage.

## Discussion

The purpose of this article was to describe the characteristics of an innovative patient navigation program in a relatively poor rural agricultural border county, and to report on the program’s success in converting cancer patients’ insurance status. In all but two instances of documented financial need, the CRCD PNP improved the patient’s insurance status. Although more data need to be collected, the CRCD PNP estimates that approximately 1 out of 5 of their patients will need financial navigation for cancer care in the future.

Insurance conversion from employer-provided private insurance to MediCal allowed the patient to receive cancer treatment without being limited to geographic location or the burden of having to pay a share of the cost. The case study illustrated the way that financial navigation often plays an important role in helping patients receive cancer treatment in a timely manner by alleviating financial burdens. In the case of Mrs. P., without financial navigation intervention, she would not have known to apply for the different categories of MediCal and would not have been able to follow the treatment plan with out-of-county oncologists. The patient had complete coverage with zero share of cost effective the first day of the month that the patient entered into the hospital. The patient was able to see any physician accepting MediCal and obtain prescriptions covered under the State MediCal plan.

CRCD financial navigation is not only beneficial for patients, it also enables the hospital and all other medical providers to receive compensation for services rendered. Financial navigation is a critical and timely service that prevents delays in receiving treatments due to noninsurance or inadequate insurance coverage. Although counselors at the hospitals provide insurance related services to patients, the complex nature of patient circumstances and limited length of hospitalization often make it difficult for them to navigate this insurance maze and successfully convert the patients’ insurance status in a timely fashion, particularly after hospital discharge. The onus then is often on the shoulders of the patient to figure out how to access treatment and pay for his/her often enormous medical bills.

Although the CRCD PNP greatly enhanced access to care for these rural, primarily Latino patients, the study is preliminary and has several limitations. The CRCD does not have the ability to measure the cost of treatment and payment to providers because those costs vary with the cancer diagnosis, stage at diagnosis, treatment plan, and many other variables. While long-term outcomes are not known, the fact that many of its patients would not have had timely cancer treatment without CRCD insurance conversion intervention and that generally, the insurance that is obtained for them is optimum for their needs, is reason to believe that there are long-term benefits.

Financial concerns are a significant thread that runs throughout the diagnostic, treatment, and post treatment journey of cancer patients. When obstacles arise, it is vital that there be an advocate/partner with the patient to mitigate or remove the financial barriers to ensure continuum of care. To that end, the CRCD PNP provides a strategic partnership with consumers in its rural border area.
